# Future of IR: Emerging Techniques, Looking to the Future…and Learning from the Past

**DOI:** 10.5334/jbsr.1727

**Published:** 2019-01-28

**Authors:** Marco Midulla, Lorenzo Pescatori, Olivier Chevallier, M. Nakai, A. Ikoma, Sophie Gehin, Pierre-Emmanuel Berthod, Romaric Ne, Romaric Loffroy, Michael Dake

**Affiliations:** 1 Center for Mini-Invasive Interventional and Endovascular Therapies - Department of Diagnostic Radiology and Image-Guided Therapies, Centre Hospitalier Universitaire de Dijon, FR; 2 Radiology Residency, Università Statale di Milano, IT; 3 Department of Radiology, Wakayama Medical University, 811-1 Kimiidera, Wakayamashi, Wakayama 641-8510, JP; 4 Department of Cardiothoracic Surgery, Stanford University School of Medicine, Falk Cardiovascular Research Center, US

**Keywords:** Interventional Radiology, Innovation, Embolization, Interventional Oncology, Image Fusion

## Abstract

Innovation has been the cornerstone of interventional radiology since the early years of the founders, with a multitude of new therapeutic approaches developed over the last 50 years. What is the future holding for us? This article presents an overview of the in-coming developments that are catching on at this moment, particularly focusing on three items: the new applications of existing techniques, particularly embolotherapy and interventional oncology; the cutting-edge devices; the imaging technologies at the forefront of the image-guidance. Besides this, clinical vision and patient relation remain crucial for the future of the discipline.

## Introduction

With the title, *Innovative solutions: an axiom of Interventional Radiology* [1], Michael Dake, a pioneer of the endovascular treatment of thoracic aortic diseases in the 1990s, addressed a short commentary regarding a case reporting the management of an exceedingly rare congenital aneurysm of the thoracic descending aorta in a premature newborn using a novel and creative once-in-a-lifetime procedure mini-invasive interventional approach, in the *Journal of Vascular and Interventional Radiology* (JVIR) back in 2012 [2]. Michael Dake stated: “That represents evidence of the magic of Interventional Radiology – a compelling and spirit-lifting reaffirmation of the timeless and beautiful truth that less is more”.

Since the early years of the discipline in the mid to late 1960s, the history of therapeutic radiology, so-called “Interventional” in 1967 by Alexander Margulis, has been an exciting way of innovation focused on the development of mini-invasive solutions for patient care. A vortical succession of events, from the first rudimental angioplasty performed in 1964 by Charles Dotter at the Oregon University in the United States, to all new therapeutic concepts, treatment modalities, and devices progressively introduced in modern medical practice (Table 1). In the 1970s balloon angioplasty was introduced by a German cardiologist working in Zurich, Andreas Gruntzig; embolizations were welcomed by a sarcastic editorial with the title “Turned-off Bleeders” in *Gastroenterology*; complex liver interventions, such as transhepatic embolizations and the concept of Transhepatic Porto-Systemic Shunt (TIPS), appeared in 1969 and were implemented in the following years for the first in-human implantation in Germany in the early 1980s. The 1980s was the decade of the stents, as three famous worldwide devices were introduced in 1985: the Palmaz, the Gianturco and the Wallstent, each named for their respective physician-inventors [3]. At the end of the century a revolution of the management of aortic disease took place, first at the thoracic level with the introduction of the stent-grafts. First invented in the mid-1980s by the vascular surgeon Nicolai Volodos in Kharkov, an industrial city of the ancient Soviet Union, (now Ukraine). Volodos’ works gained attention in occident, and the first abdominal implantations were performed in Buenos Aires, Argentina by Juan Carlos Parodi in 1990 and Nancy, France in 1994. In the mid to late 1990s, with two publications in the *New England Journal of Medicine* (NEJM), Michael Dake, then Professor of interventional radiology at the Stanford University, introduced endovascular therapy of thoracic aortic disease for aneurysms in 1994 and dissections in 1999 which universally changed the way for physicians and, mainly, patients.

**Table 1:**
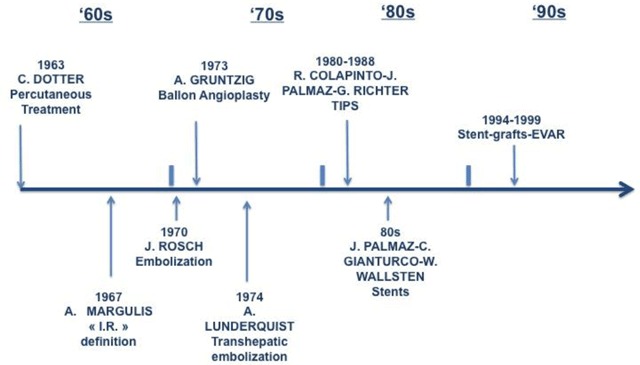
The vortical and exciting way of development of the Interventional Radiology: an incredible succession of events, from the first rudimental angioplasty performed in 1964 by Charles Dotter at Oregon University in the USA, to all new therapeutic concepts, treatment modalities, devices, progressively introduced in the modern medical practice [3].

Beyond this glorious past, interventional radiologists keep staying involved in the progressive changes of modern medicine thanks to their natural propensity to imagine new solutions for managing pathologic contexts in a mini-invasive and targeted manner. The aim of this article is to provide an overview of current developments. We identify three main items: the new fields of applications for existing techniques; cutting-edge devices; and the imaging technologies at the forefront of the image-guidance.

## New Fields of Application: Embolotherapy

### The “Emborrhoid technique”

**Figure 1:**
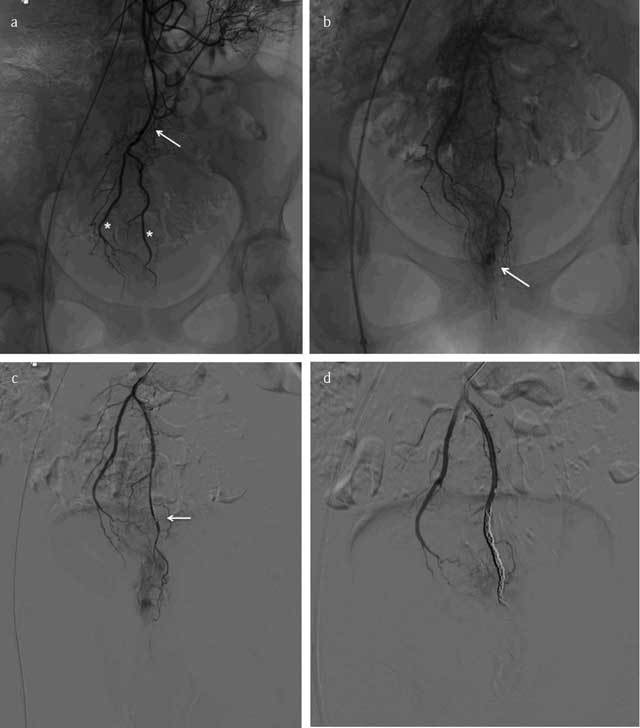
A 20-year-old woman (medical student) with history of Cloves syndrome (venous malformation at the pelvis and left leg), presenting with life threatening perineal hemorrhage from hemorrhoidal bundles: **(a)** Selective arteriography of the inferior mesenteric artery (IMA, arrow) showing vascularization of the rectum by the superior rectal arteries (SRA, asterisks). **(b)** Focal hypervascularization corresponding to the hemorrhoids. Superselective embolization of the left trunk is performed using a coaxial microcatheter (**c**, arrow) and microcoils placement **(d)** with good angiographic and clinical results. The different distal branches, respectively left, right and posterior trunk can be embolized according to the angiographic findings and anatomical classifications.

Over the last 2–3 years one of the main topics that has gained the attention of the interventional community concerns embolization therapy. The treatment of hemorrhoidal disease by transarterial embolization of the superior rectal arteries (SRA) (Figure 1), and nicely presented by Vincent Vidal and his team in Marseille under the name of “emborrhoid technique” [4], was inspired by previous experiences reporting embolization as an alternative to surgery in urgent cases of massive bleeding [5]. Ligation of the terminal branches of the superior rectal artery, to reduce the arterial supply to the hemorrhoids, is already performed in routine surgical techniques, with more or less tissue resection: the Milligan and Morgan’s hemorrhoidectomy (resection of the three hemorrhoidal cushions); the Longo procedure (resection of a ring of a ring of rectal mucosa); the more recent Elective Doppler-guided hemorrhoidal artery ligation (DG-HAL) [6,7,8]. Hemorrhoidal disease is primarily managed by conservative treatment combining hygiene and dietary measures with phlebotonics, while surgical intervention is reserved to 10% of the patients [9]. The main issue remains a prompt recovery but the complication rate ranges from 2% to 20%, with various post-operative disadvantages such as hemorrhage, stenosis, anal incontinence, and abscess. Peritonitis due to rectal perforation has also been described [10,11,12,13]. Transarterial embolization proposes a mini-invasive selective approach to achieve targeted occlusion of the arterial in-flow without recurring to an invasive surgery that requires specific equipment such as an anoscope. The first publication of the early experience in three patients has been followed by a more recent report in 2016 reporting safety and efficacy of the technique in 40 subjects [14]. Particles and coils were used as embolization agents. Early results at one month demonstrated a significant regression of the emorrhoidal bundles, absence of ischemic risk, and normal sphincteric function recovered after one month. Hospitalization and recovery time were respectively 2.5 ± 0.5 days (with 75% of the patients discharged on the first day) and 6.2 ± 0.9 days. Further prospective trials are required to better define the role of the technique beside the surgical gold standards; concurrently, applications of the emborrhoid technique in particular urgent contexts of variceal bleeding and high-risk patients are showing up in the literature and deserve attention [15,16].

### Bariatric Embolization

**Figure 2:**
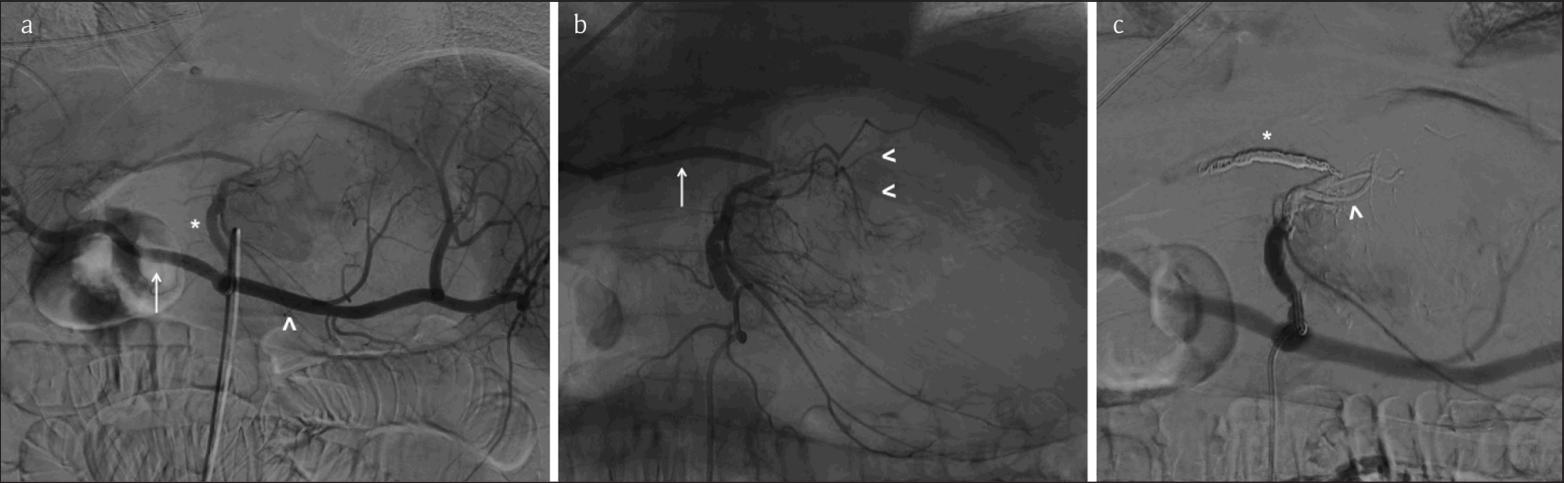
Selective arteriography of the celiac trunk (CT) in a patient addressed for massive upper bleeding from a gastric tumor. **(a)** Asterisk: left gastric artery (LGA); arrow: common hepatic artery (CHA); arrowhead: splenic artery (SA). **(b)** Superselective catheterization of the LGA showing a common anatomical variant with a left hepatic artery (LHA, arrow) originating from the LGA. On the left size, the branches supplying the lesser curvature of the stomach (arrowheads). **(c)** Embolization of the LGA is performed in this case in two steps: firstly, embolization of the LHA by coils (asterisk) to protect the liver; then, injection of a mixed oil Lipiodol-glue to obtain distal occlusion of the gastric bleeding (arrowheads). The LGA is the target in bariatric embolization for the treatment of the obesity; three ongoing trials are evaluating the outcomes of this new treatment modality, using coils and particles (see Table 1).

Another cutting-edge application of the embolization therapy in the gastrointestinal tract is the treatment of obesity. The rationale of the so-called “bariatric embolization”, introduced through an in-animal study in 2007 by a group from the John Hopkins Institute in Baltimore, is founded on the suppression of the ghrelin-producing cells, in the fundus of the stomach, by selective embolization of the left gastric artery (LGA) [17] (Figure 2). Obesity, defined as a body mass index (BMI) ≥30 kg/m^2^, is nowadays a high-impact health issue worldwide with related morbidity and mortality; it is a recognized risk factor for type 2 diabetes and several different diseases of the liver, heart, joints, lungs and even malignancies [16,17,18]. Lifestyle modifications are adopted as a first-line therapeutic approach, nevertheless the weight-loss can be obtained for 5–10% in overweight (BMI between 25 and 29.9 kg/m^2^) and obese (BMI between 30 and 34.9 kg/m^2^) patients in about three years [19,20,21,22]. Severe obesity or patients with high comorbidity can require more aggressive management by bariatric surgery, endoscopic interventions or pharmacotherapy. Embolization completes this therapeutic pattern and the clinical outcomes are under investigations. To date, there are three ongoing clinical trials focused on BAE: in the USA, the Gastric artery Embolization Trial for Lessening of Appetite Nonsurgically (GET LEAN) from Dayton Interventional Radiology at Ohio State University; the Bariatric Embolization of Arteries for the Treatment of Obesity (BEAT) carried out at Johns Hopkins Hospital; the Chinese trial NCT02786108 from Zhong-Da Hospital in Nanjing [23,24,25]. In all these studies authors are using 300–500 m or 500–710 m particles to embolize all the LGA branches and, in some case, the gastroepiploic artery (GEA). No major complications were registered while minor events, such as superficial ulcer of the mucosa at the targeted zone, was encountered. Excess body weight loss varied from 9% at three months to 17.2% at six months (with mean body weight loss among 9.2 kg). Although the actual evidence in favor of BAE is still low, more and more data support the safety and early effectiveness of this minimally invasive procedure, that in the future could be even combined with other therapeutic resources to improve the results, for instance, in severely or morbidly obese patients.

### Embolization of inflammatory joint disease

**Figure 3:**
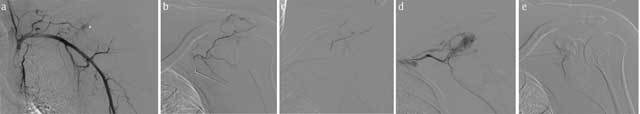
A 61-year-old man suffering from chronic osteoarthritis of the shoulder (so called “frozen shoulder”). **(a)** Arteriography of the left subclavian artery (LSA) showing the different collateral branches and a focal hypervascularization between the subacromial space and the humeral head (asterisk). Transarterial treatment by particle embolization: **(b)** selective catheterization of the thoraco-acromial artery and **(c)** control after superselective injection of 1.5 g of imipenem-cilastatin. Different articular branches (coracoid artery; anterior and posterior circumflex humeral; suprascapular) can be targeted to reduce te neo-angiogenesis related to the inflammation. In **(d)** and **(e)**, embolization of the coracoid artery, respectively before and after the particle injection. Early results of this new therapeutic concept seem promising. Courtesy of Prof. M. Nakai and Prof. A. Ikoma from the Department of Radiology, Wakayama Medical University, Japan.

A completely new field of application for embolization therapy is in the musculoskeletal system and in particular, the treatment of resistant painful conditions such as osteoarthritis, frozen shoulder (adhesive capsulitis) and overuse injuries (tendinopathy and enthesopathy) (Figure 3). The recent works of Dr. Okuno from the Musculoskeletal Intervention Center at Edogawa Hospital in Tokyo have stressed the use of the technique in these emerging indications [26,27,28,29,30]: the rationale is focused on reducing the inflammatory angiogenesis that can contribute to the chronic pain by enabling growth of new unmyelinated sensory nerves along their path. Authors report the use of the modern mini-invasive approach with three or four French radial or femoral access and assess the neovascularization of the targeted zone by digital subtraction angiography (DSA). Embolization is performed with both pharmacological agents and calibrated microspheres (imipenem/cilastatin sodium, IPM/CS; 10–70 m particles). Results in terms of pain relief seem promising, thus endovascular therapy could gain a new role in the management of those conditions where no consensus exists, for instance the frozen shoulder.

## The Next Step of Interventional Oncology: Interventional Immuno-oncology

**Figure 4:**
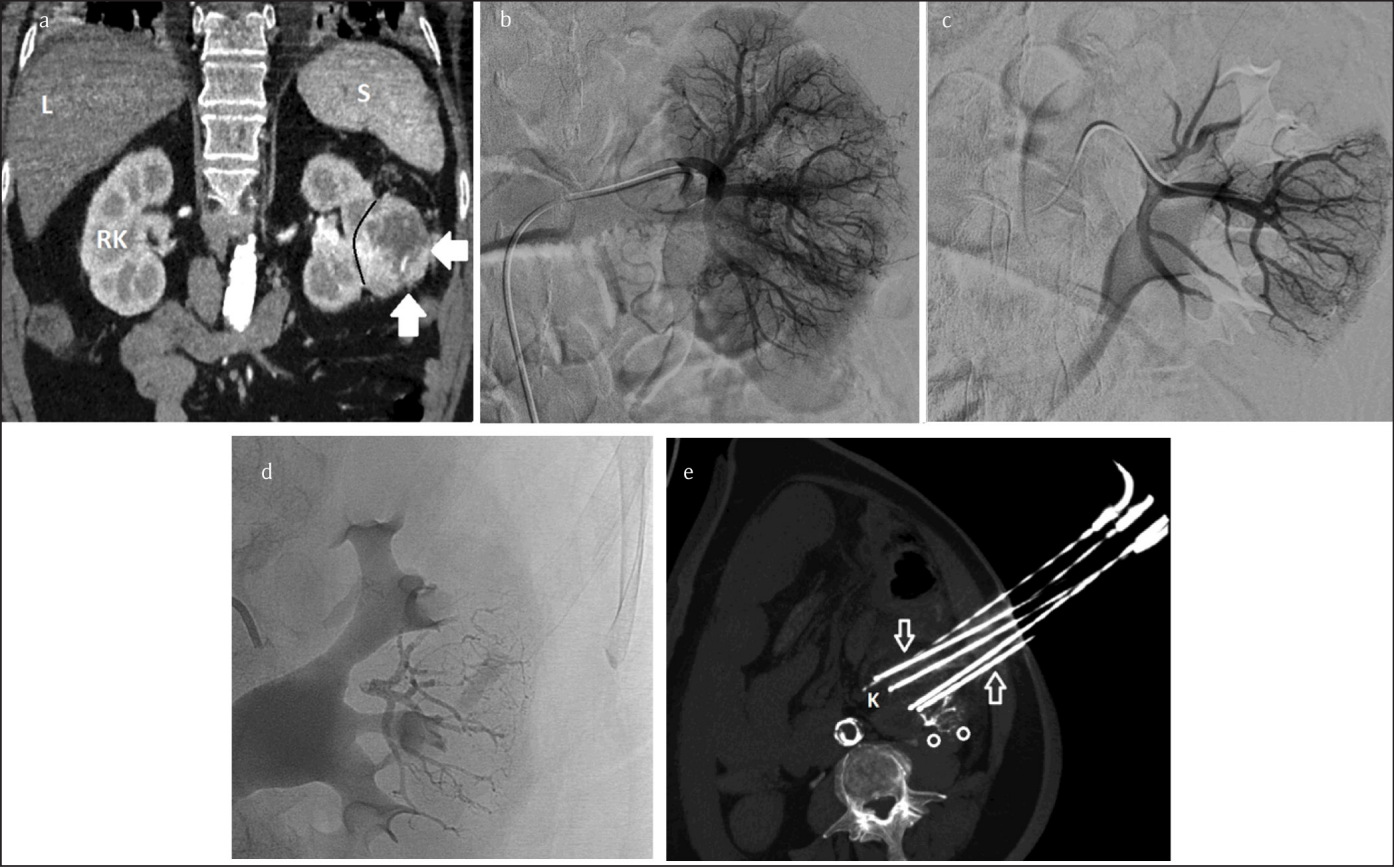
Interventional management of a renal mass in an inoperable patient, combined approach: transarterial preoperative embolization is performed through femoral access **(b)** using selective catheterization **(c)** and injection of glue **(d)**, thus obtaining devascularization before percutaneous ablation. **(e)** CT-guided cryotherapy; combination of the percutaneous ablative therapies and the immune-modulating drugs in the so-called “Interventional Immunooncology” becomes an unexplored field of application with a tremendous potential to investigate in the next future.

In 2006 the World Conference of Interventional Oncology took place in Cernobbio in front of the picturesque view of the Lake Como in northern Italy, and a new field of medicine was born. At that time, the term “Interventional Oncology” assembled the different techniques and new therapeutic concepts for cancer that had been developing with Interventional Radiology under a discipline that was subsequently integrated into the modern approach for oncologic patient care. We are aware nowadays of the role of the percutaneous ablation therapies and chemo- and radio-embolizations in the therapeutic algorithms for cancer care. In the last few years a more innovative concept is appearing, well explained in some recent cutting-edge reviews published in radiologic and clinical journals [31,32,33]: the interventional immuno-oncology (IO). Curiously, the acronym IO is already used by both radiologists and oncologists to refer respectively to interventional radiology and immuno-oncology. The meeting between these two disciplines is becoming a promising field of innovation for the mini-invasive, patient-specific approach to neoplastic pathology therapy, able to maximize the efficacy of percutaneous tumor ablation therapies and improve the systemic immunologic response to the residual neoplastic cells. The phenomenon, whereby a locally applied therapy triggers a distal antitumor response, is termed the abscopal effect [31,33]. The different techniques, radiofrequency (RF), microwave (MWA), cryoablation, focused ultrasound (FUS), and embolization, variably induce tissue necrosis, thus exposing the tumoral antigens to the immune system and stimulating the physiologic immune response. Specific in-animal trials have assessed the potential of each ablative therapy for interacting with the immunologic processes [34,35,36,37,38]; for instance, among the different ablative percutaneous solutions, cryoablation seems to have the best potential, so much so that some authors have referred to it in terms of “in-vivo dendritic cell vaccine” to highlight the effective impact on the immunologic mechanisms. Several different elements (intracellular organs, antigens, damage-associated molecular patterns DAMPs) are released after the tumoral necrosis and phagocytized by the dendritic cells (DC) activating the complex pathways of the immune response (nuclear factor kappa-light-chain-enhancer of activated B cells, NF-κβ; Heat Shock Protein 70; chemokines, like the Monocyte Chemoattractant Protein-1, CXCL16; cytokines like the TNF-α, IL-1, & IL-16; danger signals such as ATP, cal-reticulin, HMGB1). Oncologists have already adopted immunotherapy to activate and enhance the physiologic response; immuno-modulating drugs are largely divided in two classes, respectively targeting the innate immune system and the adaptive response. New pharmaceuticals are, for instance, the inhibitors of the “check-points” that prevent the inappropriate activation of a cell-mediated immune response (adaptive), such as the Ipilimumab used in metastatic melanoma, the PD-1 inhibitors (Pembrolizumab, Nivolumab, Durvalumab, Avelumab) approved for the treatment of melanoma, renal cell carcinoma, bladder cancer, non-small cell lung cancer, Hodgkin lymphoma, Merkel cell carcinoma and solid tumors. Combination of these new drugs targeting both the adaptive and innate immune system with ablative treatments has been tested in animal models, showing improvement of mean survival, cytolytic activity, tumor-specific T cell activation and dendritic cell maturation. The progressive advances in the understanding of the interactions between interventional therapies and the immune response will advance future clinical applications of interventional immuno-oncology and offer an exciting field of innovation for interventional radiologists (Figure 4).

## Innovative Devices: Vascular

### Dialysis Access

During the last three years, two percutaneous devices have been developed, thereby introducing a new concept of the percutaneous arteriovenous fistula (AVF) creation. As is well known, AVF provide the best vascular access for hemodialysis in patients with end-stage renal disease, with low risk of infections and a better cost-benefit ratio compared to catheters and grafts. Surgical technique is standardized since long time and the innovation would substantially provide the advantage to avoid incision. The systems commercially available use a single or double catheter approach respectively, while also using a RF tool to create the fusion channel between the arterial and venous wall. The thermal resistance anastomosis device (TRAD) is a single-catheter system creating the AVF by direct puncture of the deep communicating vein (DCV) and the proximal radial artery (PRA) under ultrasound (US) guidance. Angioplasty of the new shunt is performed with a 4–5 mm balloon. Results published in 2018 for 34 patients showed a primary and primary assisted patency rate as expected, of 82% and 94% respectively with successful maturation of the AVF (diameter > 6 mm for 10 cm, flow > 600 mL/min) [39,40,41]. These positive outcomes have been also confirmed by the US multicenter pivotal trial [41]. The second device consists of a pair of over-the-wire 6-F catheters using magnetic force aligned in the ulnar vein and artery. Connection is accomplished by a RF cutting current that vaporizes the tissue. The results of the prospective, multicenter Novel Endovascular Access Trial (NEAT) have been presented this year (2018) for 80 patients showing a success rate of 98%, a primary efficacy endpoint of 87% (% of endoAVFs physiologically suitable for hemodialysis within three months of creation; freedom from fistula stenosis and thrombosis and brachial artery flow ≥500 mL/min and vein diameter ≥4 mm) and a patency rate at 12 months of 69% [42]. These outcomes are in favor of a valuable new mini-invasive modality for the creation of functional AVF with low complication rate.

## Image-guidance Technologies

### Virtual Reality

Since the time of fluoroscopy, the panel of techniques and methods used to guide percutaneous interventions has evolved tremendously. A real revolution has been introduced over the last decade by the multimodality image fusion concept that has definitely gained major attention in the modern scenario of the interventional radiology. The use of the cone-beam CT (CBCT) in combination with preoperative imaging has become an invaluable tool to accomplish complex endovascular repair and improve embolization and interventional oncology practice. More recently, cutting-edge technology available in commercial products such as the information-communications field and video games, is advancing extended reality in medical use. Elchanan Bruckheimer, a pediatric cardiologist from Israel introduced first the application of this new technology in clinical medical imaging [43], as shown in an episode of the famous American TV series *Grey’s Anatomy*. Extended reality generally defines the spectrum or “virtuality continuum” of the interaction between the observer and a surrounding environment artificially modified through a wide range of digital creations. As shown in Table 2, augmented reality (AR) and virtual reality (VR) stay at the two ends of the spectrum respectively, with insertion of 2D or 3D digital images in an “untouched” native environment (AR) and creation of a completely new synthetic scenario (VR) [44].

**Table 2:**
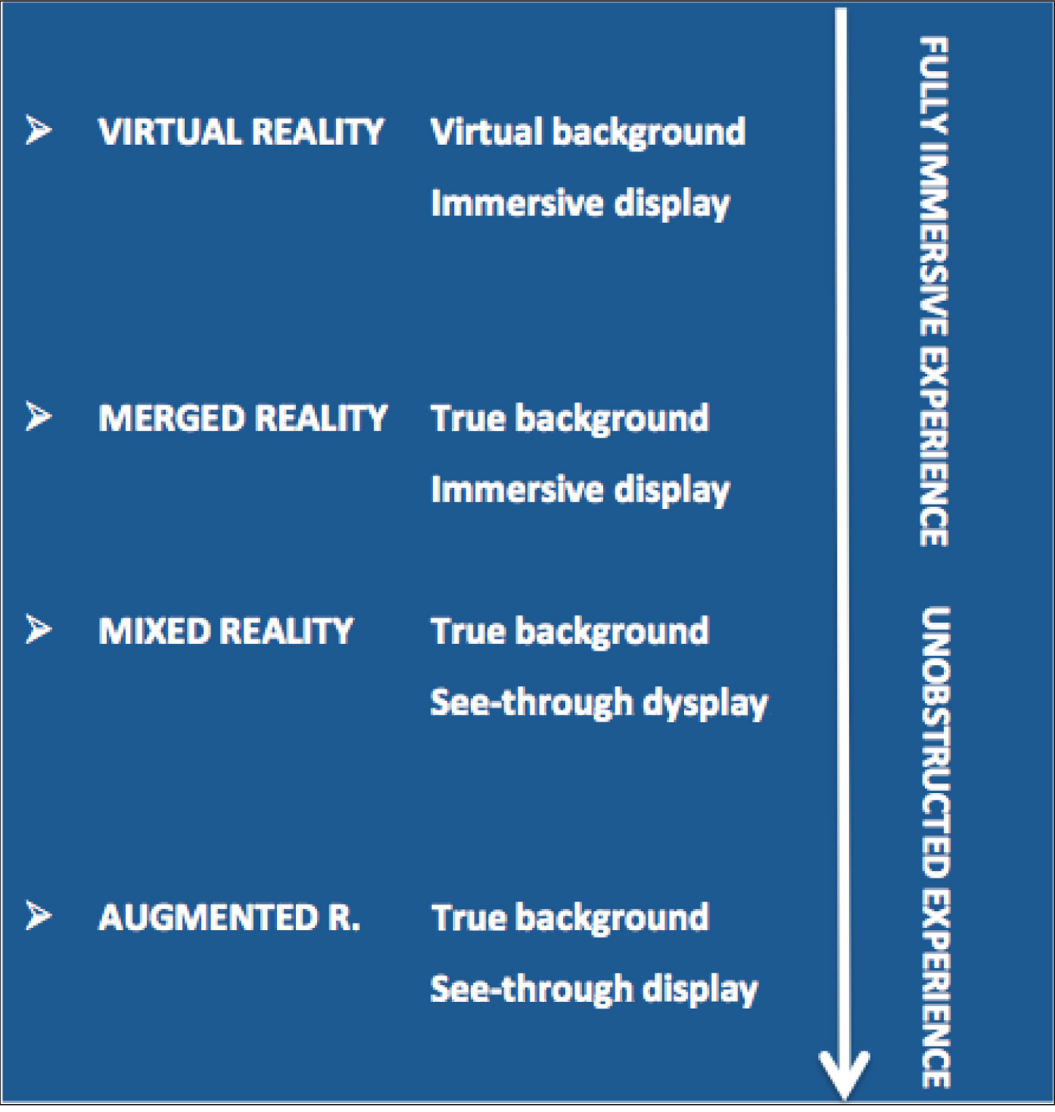
The large spectrum of the Extended Reality, involving different type of virtual experiences where the observer interacts with the surrounding environment. Merged (MeR) and Mixed Reality (MxR) are the most adapted concepts for medical applications as they allow interaction with digital objects while preserving contact with the real surrounding environment (patient and operatory theatre).

The potential introduction of the extended reality (from augmented reality to virtual reality) is a new horizon that can integrate the multimodality guidance and is gaining attention of new start-ups and commercial companies. Merged (MeR) and Mixed Reality (MxR) are the most adapted concepts for medical applications as they allow interaction with digital objects while preserving contact with the real surrounding environment. Clinical applications include intraprocedural visualization of patient’s data, 3D holographic reconstruction of organs anatomy for virtual interactive assessments and training purposes, patient and family preoperative information.

## Final Considerations: Looking to the Future… and Learning from the Past

Imagination-innovation, lower impact on the patient and mini-invasive approach have been, and still remain, the cornerstones of our discipline. Interventional radiologists have the merit of being at the forefront of the modern medicine thus, as treated above, a wide panel of therapeutic options is nowadays part of standard algorithms of care. Technical advances are exciting and intrigue every physician particularly if he/she is an interventional radiologist. Nevertheless, the primum movens must be centered on a main issue: the patient. Interaction with people, that is seeing patients before and after the worst or best procedure, is mandatory; building outpatient clinic for direct referral to interventional radiology should be part of our practice; clinical vision and multidisciplinary discussion remain crucial for recognition of the specialty.

This, in our opinion, is the third cornerstone of interventional radiology and maybe the more innovative approach of our decade.
